# Antimicrobial Activity of Nitrogen-Containing 5-α-Androstane Derivatives: In Silico and Experimental Studies

**DOI:** 10.3390/antibiotics9050224

**Published:** 2020-04-30

**Authors:** Lela Amiranashvili, Nanuli Nadaraia, Maia Merlani, Charalampos Kamoutsis, Anthi Petrou, Athina Geronikaki, Pavel Pogodin, Dmitry Druzhilovskiy, Vladimir Poroikov, Ana Ciric, Jasmina Glamočlija, Marina Sokovic

**Affiliations:** 1 TSMU I. Kutateladze Institute of Pharmacochemistry, P. Sarajishvili str. 36, Tbilisi 0159, Georgia; l.amiranashvili@tsmu.edu (L.A.); n.nadaraia@tmsu.edu (N.N.); m.merlani@tmsu.edu (M.M.); 2 University of Patras, 26500 Patra, Greece; kamoutsi@upatras.gr; 3 School of Pharmacy, Aristotle University of Thessaloniki, 54124 Thessaloniki, Greece; anthi.petrou.thessaloniki1@gmail.com; 4 Institute of Biomedical Chemistry, 119121 Moscow, Russia; pogodinpv@gmail.com (P.P.); dmitry.druzhilovsky@ibmc.msk.ru (D.D.); vladimir.poroikov@ibmc.msk.ru (V.P.); 5 Mycological Laboratory, Department of Plant Physiology, Institute for Biological Research “Siniša Stanković”, University of Belgrade, 11060 Beograd, Serbia; rancic@ibiss.bg.ac.rs (A.C.); jasna@ibiss.bg.ac.rs (J.G.); mris@ibiss.bg.ac.rs (M.S.)

**Keywords:** antibacterial activity, antifungal activity, potency against the resistant strains, PASS, GUSAR, CLC-Pred, molecular docking, UDP-*N*-acetylenolpyruvoylglucosamine reductase inhibition, 14*α*-demethylase inhibition

## Abstract

We evaluated the antimicrobial activity of thirty-one nitrogen-containing 5-α-androstane derivatives in silico using computer program PASS (Prediction of Activity Spectra for Substances) and freely available PASS-based web applications (such as Way2Drug). Antibacterial activity was predicted for 27 out of 31 molecules; antifungal activity was predicted for 25 out of 31 compounds. The results of experiments, which we conducted to study the antimicrobial activity, are in agreement with the predictions. All compounds were found to be active with MIC (Minimum Inhibitory Concentration) and MBC (Minimum Bactericidal Concentration) values in the range of 0.0005–0.6 mg/mL. The activity of all studied 5-α-androstane derivatives exceeded or was equal to those of Streptomycin and, except for the 3β-hydroxy-17α-aza-d-homo-5α-androstane-17-one, all molecules were more active than Ampicillin. Activity against the resistant strains of *E. coli, P. aeruginosa*, and methicillin-resistant *Staphylococcus aureus* was also shown in experiments. Antifungal activity was determined with MIC and MFC (Minimum Fungicidal Concentration) values varying from 0.007 to 0.6 mg/mL. Most of the compounds were found to be more potent than the reference drugs Bifonazole and Ketoconazole. According to the results of docking studies, the putative targets for antibacterial and antifungal activity are UDP-N-acetylenolpyruvoylglucosamine reductase and 14-α-demethylase, respectively. In silico assessments of the acute rodent toxicity and cytotoxicity obtained using GUSAR (General Unrestricted Structure-Activity Relationships) and CLC-Pred (Cell Line Cytotoxicity Predictor) web-services were low for the majority of compounds under study, which contributes to the chances for those compounds to advance in the development.

## 1. Introduction

According to the World Health Organization (https://www.who.int/news-room/fact-sheets/detail/the-top-10-causes-of-death), infectious diseases are among the top ten leading causes of death worldwide. This is mainly due to the emerging antimicrobial resistance, which is a threat to global health itself (https://www.who.int/news-room/feature-stories/ten-threats-to-global-health-in-2019). In particular, nosocomial infections caused by methicillin-resistant *Staphylococcus aureus* (MRSA), vancomycin-resistant *Enterococcus faecium* (VRE), and drug-resistant *Streptococcus pneumoniae* have been designated as severe public threats by the US Centers for Disease Control and Prevention [1]. Furthermore, the resistant bacteria capable of surviving in the presence of the almost all known antibiotics, such as multidrug-resistant Staphylococcus aureus (MRSA), are the major source of concerns worldwide [2,3,4,5,6,7]. It is essential to outline the main factors contributing to the antimicrobial resistance to find a way to deal with it. The main reasons why bacteria can acquire and demonstrate resistance in the clinic are as follows: (1) high rates of mutations (in some bacteria); (2) exchange of genetic information via mobile genetic elements (plasmids) in some bacteria; (3) violation of medical prescriptions for taking antibiotics; (4) a limited number of antimicrobial agents in clinical practice.

Therefore, new approaches are needed to fight antimicrobial resistance. Both modifications of known and discovery of novel antibacterial and antifungal molecules are applied to develop the antimicrobial agents active against the resistant pathogens [8,9,10,11,12,13].

One of the promising strategies is the chemical modification of the steroids. Two of the adopted ways of doing so are the introduction of the oxime group in the steroid scaffold and attachment of amino groups to steroids. Previously, it was shown that such modifications improve many biologically relevant properties of steroids: modified derivatives are often less toxic and possess the pleasant bioavailability. Moreover, many such compounds were shown to be active against the bacteria, including resistant ones [14,15,16,17,18,19,20]. Also, steroidal oximes [21,22], and azides [23,24,25] are considered as the suitable starting points for the development of more complex molecules having their advantages [26,27,28,29,30,31].

It is worth to notice that we found strong structure-activity relationships for antiarrhythmic and radioprotective activity (RPA) of epimeric 3-amino-5α-androstan-17-ol and 17-amino-5α-androstan-3-ole. 17β-Amino-5α-androstan-3 β-ole is characterized by the best antiarrhythmic activity and 3α-amino-5α-androstan-17α-ole with the best RPA [21]. 3α-Amino-5α-androstan-17α-ole was selected and evaluated for antibacterial and antifungal activity. Results proved the high antimicrobial activity of this epimer [22]. According to our previous studies on the *N*-containing derivatives of 5α-androstane series, the presence of 3α-amino- and 17α-hydroxy functional groups for antimicrobial and radioprotective activity [21,22] is essential. Recently, we found that the antimicrobial action of *N*-containing 5α-androstane derivatives is probably due to the very selective interaction since even slight changes in the molecular structure may reduce or increase their activity significantly [22].

These data [23,24] prompted us to continue study in this field and investigate the antimicrobial activity of 17-amino-5α-androstan-3-oles and derivatives as well as intermediate *N*-containing compounds that we have synthesized earlier.

Thus, the purpose of our study was in silico evaluation of the antimicrobial potential of thirty-one nitrogen-containing 5-α-androstane derivatives and further experimental testing of their antibacterial and antifungal activity, including action on the resistant strains. Thirty-one amino-, amido-, hydroximino-, phtalimido-, d-homo-, and azido-steroidal derivatives 1–27, that we synthesized earlier [21,32,33,34,35,36,37,38,39,40,41,42,43], were prepared and evaluated for antimicrobial actions.

## 2. Results and Discussion

### 2.1. Chemistry 

**Scheme 1:**
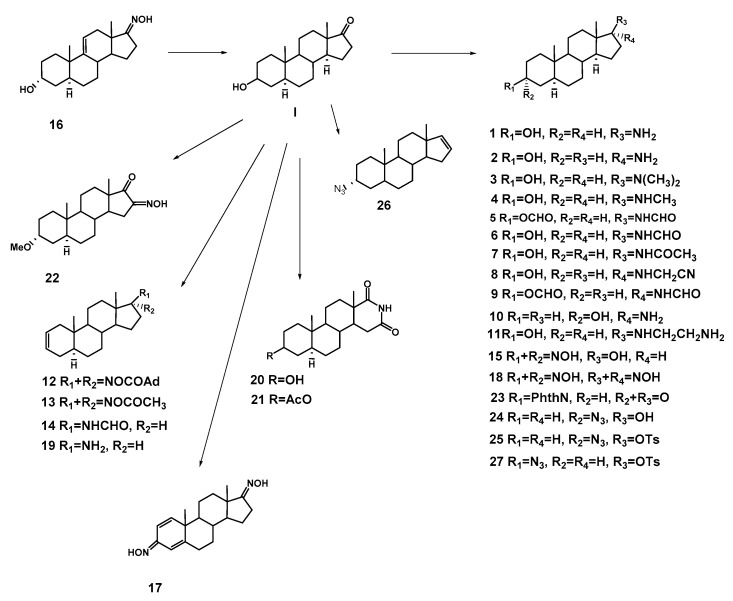
Synthetic routes of compounds **1**–**27**.

In continuation of our studies of *N*-containing 5α-androstane derivatives we conducted further in silico and in vitro studies of their antimicrobial activity and its selectivity [22]. Most of the compounds synthesized earlier revealed different pharmacological effects. According to our previous studies on the *N*-containing derivatives of 5α-androstane series, the importance of 3α-amino- and 17α-hydroxy functional groups for antimicrobial and radioprotective activity [21,22] was shown.

d-homoandrostane derivatives **20** and **21** were synthesized from oxime **22** using Beckmann molecular rearrangement procedure [42] and 3α-phthalimido-5α-androstane-17-one **23** from epiandrosterone by Mitsunobu reaction [43]. The data about any biological activity of steroidal oximes **15** and **22**, phthalimido- **23**, and azido steroids **24** and **27** have not been found so far in the literature. The structure of compounds is presented in Table 1 and way of their preparation in Scheme 1. The results of biological testing provide a strong impetus for a more extensive study of the derivatives mentioned above and the continuation of the search for new, highly effective antimicrobial agents among *N*-containing 5α-steroidal compounds.

Compounds **28**–**30** were synthesized according to the procedure described earlier [44,45,46]; compound **31** was purchased from Fluka.

### 2.2. Biological Activity and Toxicity Predictions

PASS prediction of antimicrobial activities was performed for thirty-one compounds selected for investigation. Antibacterial activity was predicted for 27 out of 31 compounds with probability “to be active” Pa values ranging from 0.298 to 0.458. Antifungal activity was predicted for 25 compounds with Pa values ranging from 0.171 to 0.427 (Table 2).

Pa values below 0.5 indicate not only the probability for the chemical compound to be found active in the experiment, but also testify on its relative novelty to the training set or the presence of similar compounds among the ones having activities besides predicted, which is probably the case for steroids, known for their wide range of biological activities [47,48].

PASS predicts the antibacterial and antifungal effects for chemical compounds in general, furthermore, also activity against the limited number of bacteria and fungi. In addition, to rationally select the particular bacterial and/or fungal target for chemical compound, AntiBAC-pred [49,50] and AntiFun-Pred [51] may be used, since they are able to predict activity against many distinct bacterial and fungal species and strains. AntiBac-Pred and AntiFun-Pred differ from standard version of PASS in training sets, which consist only of the structures of chemical compounds evaluated experimentally against bacteria (AntiBac-Pred) or fungi (AntiFun-Pred).

Application of the AntiBac-Pred to chemical structures of the studied 5-α-androstane derivatives provided the following results: 23 out of 35 compounds were predicted as active ones against the *L. plantarum* and *S. lugdunensis*; 4 compounds were predicted as active against *B. sphaericus*, *C. ramosum*, *P. gingivalis*, resistant *S. simulans*, and *S. mutans.* Besides, at least one compound has been predicted as active against one or more of 25 other bacteria, including two resistant strains (resistant *S. simulans* and resistant *M. ulcerans*).

AntiFun-Pred predicted activity for the 27 out of 31 compounds on 18 different fungi, including *Candida albicans* (13 compounds were predicted to be active), *Saccharomyces cerevisiae* (12 compounds), *Absidia corymbifera* (10 compounds), *Rhizopus oryzae* (9 compounds), and *Mucor hiemalis* (8 compounds).

Therefore, the compounds under study may be tested experimentally against the vast and diverse set of microbial organisms. The results of prediction, including up to three best-rated chemical structures for the selected bacteria and fungi, are given in Supplementary Materials.

Predictions of rat acute toxicity for intraperitoneal and oral routes of administration obtained using computer program GUSAR [52,53,54] are given in the Supplementary Materials. As could be seen from this data, all analyzed compounds belong to the class five or four of the hazard according to the OECD classification [55].

CLC-Pred [49,50], one more PASS-based web resource, was used to assess the potential cytotoxicity of the studied compounds against the 22 non-tumor cell lines. 22 out of 31 compounds were not predicted as cytotoxic at the cutoff Pa > 0.5 (Supplementary Materials). Compounds **12**, **18**, and **17** were predicted as cytotoxic against the HUVEC cell line with Pa = 0.79, 0.77, and 0.70, respectively. Compound **30** was predicted as cytotoxic against MOLT-4 and MDA-MB-468 with Pa = 0.63 and 0.57, respectively. Compounds **1**, **2**, and **10** were predicted as cytotoxic against the WI-38 VA13 cell line, and compound **16**, as cytotoxic against the HUVEC cell line, with Pa about 0.52. One may also select the other compounds with a low probability of cytotoxic effect as the most promising in the terms of the safety for further studies (e.g., for compounds **8**, **18**, **31**, etc. Pa < 0.3).

It is necessary to notice that the PASS-based approach estimates the probability of belonging to the classes of “actives”. However, it does not determine the concentration/dose, which will induce the predicted action. Therefore, the dose–cytotoxic effect relationships should be studied for the compounds mentioned above, particularly against the predicted vulnerable cell lines.

Overall, PASS and PASS-based web applications are able not only to provide the computational assessment for chemical compounds to have general antimicrobial effect and activity against the particular microbial species and strains, but also to give some insights about cytotoxicity against the particular non-tumor cell lines.

### 2.3. Biological Evaluation

#### 2.3.1. Antimicrobial Activity

The antimicrobial activity of the synthesized compounds was evaluated using the microdilution method for determining the minimal inhibitory and minimal bactericidal/fungicidal concentrations. 

Results of evaluation of antibacterial activity of compounds **1**–**31** are shown in Table 3. The order of activity can be presented as follows: **19** > **10** > **1** > **2** > **4** > **11** > **3** > **26** > **22** > **28** > **30** > **5** > **15** > **16** > **23** > **20** > **29** > **14** > **7** > **24** > **13** > **12** > **8** > **9** > **31** > **17** > **18** > **21** > **6** > **25** > **27**. 

The best antibacterial activity was exhibited by compound **19** with MIC at 0.0005–0.007 mg/mL and MBC at 0.0007–0.015 mg/mL. In contrast, compound **27** showed the lowest activity with MIC and MBC at 0.3 mg/mL and 0.60 mg/mL against *E. coli.* Compounds **6** and **25** were inactive against strains.

It should be noticed that bacteria, in general, showed different sensitivities to compounds tested. Nevertheless, three bacteria species, *S. aureus, L. monocytogenes,* and *P. aeruginosa* appeared to be very sensitive to compound **19**. Completely different was the sensitivity of *E. coli* and *S. typhimirium* toward compounds tested. Thus, the antibacterial potency against *S. aureus* can be presented as: **19** > **1** = **3** = **4** = **10** > **2** > **11** > **22** > **28** > **5** = **14** = **16** = **20** = **23** = **30** > **8** = **24** > **18** > **29** = **31** > **6** = **7** = **9** = **12** = **13** = **15** = **17** = **21** = **25** = **26** = **27**, while against *L. monocytogenes* as: **19** > **3** = **10** > **1** > **2** = **4** > **28** = **29** = **30** > **5** = **11** = **22** > **8** > **20** > **15** = **16** > **7** = **14** = **23** = **24** = **31** > **21** > **9** = **12** = **17** = **18** > **6** = **13** = **25** = **26** = **27.** *S. aureus* was not sensitive to twelve compounds, while *L. monocytogenes* appeared to be more sensitive to compounds tested being not sensitive only to five compounds (**6, 13, 25, 26,** and **27**).

As far as Gram-negative bacteria are concerned, the potency against *S. typhimirium*, the most sensitive bacterium, can be presented as follows: **19** > **3** = **4** = **10** > **1** > **2** > **5** = **11** = **24** = **30** > **20** >= **16** = **28** = **29** > **7** = **8** = **14** = **15** = **23** > **9** > **17** = **18** = **21** = **22** > **31** > **6** = **12** = **13** = **25** = **26** = **27,** while against the most resistant among all bacteria tested *P. aeruginosa* as: **19** > **4** > **11** = **20** = **28** = **29** >= **2** >= **1** = **10** > **5** >= **22** = **24** = **26** = **30** > **16** > **14** = **15** = **18** = **31** > **23** > **8** >= **13** = **17** > **7** = **9** = **12** > **3** > **6** = **25** = **21** = **27**.

Completely different was the sensitivity of *E. coli* and *S. typhimirium* to compounds tested. As it is already mentioned, compound **19** exhibited excellent activity against all bacterial strains tested. On the other hand compounds **19, 1, 3, 4,** and **2** exhibited good activity also against *S. aureus* and *L. monocytogenes* with MIC and MBC at 0.0005–0.02 mg/mL and 0.0007–0.04 mg/mL respectively, while some of them (**19, 2, 4**) with MIC at 0.0015–0.025 mg/mL and MBC 0.003–0.037 mg/mL against *P. aeruginosa* and **1, 3, 4,** and **19**, with MIC and MBC at 0.005–0.02 and 0.007–0.037 mg/mL respectively against *S. typhimirium*. Good activity against this bacterium was observed also for compound **5**. Compounds **1, 4, 10,** and **28** exhibited good activity against *E. coli* with MIC at 0.007–0.025 and MBC at 0.015–0.075 mg/mL.

In particular, for the Gram-positive bacteria, the range of MIC and MBC were at 0.0005–0.3 mg/mL and 0.0007–0.45 mg/mL, respectively, while for the Gram-negative bacteria, MIC and MBC ranged at 0.0015–0.3 and 0.003–0.6 mg/mL. It seems that Gram-positive bacteria are more sensitive to the tested compounds than Gram-negative bacteria. 

At the same time, it was observed that many compounds exhibited the same potency among the same bacteria species. Thus, for example, compounds **1**, **3**, and **22**, as well as **4** and **10**, have the same potency against *S. aureus*. Compounds **2** and **4**, as well as **28** and **29**, showed the same activity against *L. monocytogenes*. The same was observed for other species as well. On the other hand, some compounds appeared to be inactive against some bacteria species. Thus, compounds **6**, **7**, **9**, **12**, **13**, **15**, **17**, **21**, **25**, **26**, and **27** did not display any activity against *S. aureus* being active against almost all other species. Compounds **6**, **13**, **25**, **26**, and **27** were also inactive against *L. monocytogenes*.

In general, compounds **6** and **27** were found to be the most inactive compounds. It should be mentioned that compounds **1**–**4, 10, 11, 28**, and **29** showed better antibacterial potency than both antibiotics used as reference drugs.

A structure-activity relationship analysis revealed that 17β-amino substituent of 5α-androst-2-en (**19**) is favorable for antibacterial activity, while replacement 17-β-amino substitution with 17-acetoximino (**13**) as well as adamantoyloximino group (**12**) was very negative. 

For 5α-androstan derivatives the presence of 17α-amino-3α-hydroxy- (**10**), as well as 17β-amino-3β-hydroxy groups (**1**) is beneficial for antibacterial activity. In general, replacement of the 17β-amino with 17α-amino group (**2**) as well as of alkyl substitution of 17β-amino group led to compounds **4** and **11** with decreased, but still good activity. Introduction of 17β-formamido group led to compound **6** which was completely inactive against all bacteria tested. In general, he order of activity of these derivatives can be presented as **10** > **1** > **2** > **4** > **11** > **3** > **8** > **7** > **6**.

For hydroximino-androstan derivatives (**15, 16, 17, 18, 22)**, the order of activity can be presented as follows: **22** > **15** > **16** > **17** > **18.** Thus, the most beneficial appeared to be the presence of 3α- methoxy- and 16-hydroximino groups in 5α-androstan-17-one cycle (**22**), as well as 3-hydroximino-17-hydroxy groups in 5α-androstan core (**15**), while the introduction of 3,17-hydoximino groups in androsta-1,4-dien core **(17**) as well as in 5α-androstane core (**18**) was detrimental. The introduction of two hydroximino groups in positions 3 and 17 in the 5α-androstan (**18**), as well as in the androstan-1,4-dien cores (**17**) appeared to be very negative for antibacterial activity. Thus, the most beneficial for activity in this group was the presence of 3α-methoxy- and 16-hydroximino- in 5α-androstane-17-one core (**22**) while introduction of 3,17-hydoximino groups in androsta-1,4-dien core (**17**) as well as in 5α-androstane core (**18**) was detrimental. 

Finally, the lowest activity among all compounds tested was observed in case of 17β-tosyloxy- as well as 3α- and 3β-azido 5α-androstan derivatives (**25**, **27**).

It should be mentioned that in general, azido derivatives, together with hydroxyimino derivatives, were among the less active steroids.

In conclusion, the structure–activity relationship studies revealed that beneficial for antibacterial activity is the presence of the 17β-amino group in the 5α-androst-2-en core (**19**) and 17β-amino-3β-hydroxy group in 5α-androstan (**1**) and also 3α-methoxy-16-hydroximino substituents in 5α-androstan-17-one core (**22**) as well as 3β-hydroxy group in 17a-aza-d-homoandrost-5-en-17-one cycle (**28**).

All compounds were tested against three resistant bacterial strains (MRSA, *P. aeruginosa* and *E. coli)* (Table 3) and their antibacterial potential can be presented as follows: **19** > **1** > **22** > **2** > **28** > **24** > **10** > **11** > **5** > **3** > **16** > **4** > **30** > **7** > **18** = **21** > **20** > **15** > **26** > **9** > **14** > **12** > **29** > **8** = **13** > **23** = **25** = **31** > **17** > **6** = **27**. 

Compound **19** again showed the best activity as in the case of ATCC bacteria with MIC and MBC at 0.000015–0.015 mg/mL and 0.0003–0.037 mg/mL, respectively. The lowest antibacterial activity was observed for compound **27** with MIC 0.30 mg/mL and MBC 0.60 mg/mL.

The resistant strains, as in case of the non-resistant strains, expressed different sensitivity-towards compounds tested as well. Nevertheless, all three resistant strains were susceptible to **19** and very resistant to **27**.

The structure–activity relationship study revealed that as in the case of non-resistant bacteria, the 17β-amino substituent of 5α-androst-2-en (**19**) is favorable for antibacterial activity. The presence of 17β-amino-3β-hydroxy-(**1**) as well as 3α-methoxy-16-hydroximino groups (**22**) in 5α-androstan- and 5α-androstan-17-one cores appeared to be beneficial too.

In a group of 5α-androstan-3β-ol derivatives, the most beneficial for antibacterial activity against resistant strains was the presence of the 17β-amino group (**1**). Epimerization to 17α-amino **(2**) decreased a little activity. The replacement of the 3β-hydroxy group in compound **2** by 3α-hydroxy resulted in less active compound **10**. In general, the substitution of the free 17-amino group was not beneficial for activity against resistant strains. Thus, the presence of 17β-*N*-methylamino- (**4**), as well as 17α-cyanomethylamino groups (**8**), appeared to be very negative for activity.

For 5α-androst-2-en derivatives, the most beneficial was the presence of the 17β-amino group (**19**). This compound, in general, was the most active among all 31 compounds tested. The positive influence also had 17β-formamido substituent (**14**), while acetoximino- (**13**) had a negative effect on antibacterial activity against resistant strains. In a group of hydroximino derivatives, the best result was observed with 3α-methoxy-16-hydroximino substitution of 5α-androstan-17-one core (**22**), which showed, in general, good activity among all compounds tested. On the contrary, the presence of a 3,17-hydroximino group and two double bonds 1,2 and 4,5 in A ring of steroid core (**17**) was very negative. Among azido derivatives, the most positive contribution to the activity was shown by the presence of 3α-azido-17β-hydroxy groups in 5α-androstan ring (**24**). The substitution of the 17β-hydroxy group by tosyloxy was detrimental for the activity **(25**). Finally, for 17a-aza-d-homoandrost-5-en-17-one **28** derivatives beneficial for activity was the presence of 3β-hydroxy group as well as the double bond in 5,6 positions (**28**). The reduction of this double bond had a negative effect leading to compound **29**, which is among the less active compounds.

In conclusion, the structure–activity relationship studies against resistant strains revealed that substituents beneficial for antibacterial activity appeared to be the same as in case of non-resistant bacteria. 

#### 2.3.2. Evaluation of Antifungal Activity

The antifungal potential of tested compounds is shown in Table 4 and can be presented as: **28** > **19** > **3** > **11** > **15** > **1** = **16** > **10** > **29** > **9** > **4** > **14** > **13** > **23** > **12** > **24** > **5** > **22** > **18** > **2** > **7** > **8** > **25** > **17** > **26** > **21** > **6** > **27** > **31** > **30**. 

Compound **28** exhibited the best antifungal activity with MIC at 0.03–0.037 mg/mL and MFC at 0.007–0.075 mg/mL. The lowest antifungal potency was observed for compound **30** with MIC and MFC at 0.20–2.40 mg/mL and 0.30–2.40 mg/mL respectively.

Ketoconazole displayed antifungal activity at MIC 0.15–1.0 mg/mL and MFC at 0.20–1.50 mg/mL, while bifonazole at MIC 0.10–0.20 mg/mL and MFC at 0.20–0.25 mg/mL. From the observed results, it is obvious that all compounds are more potent than both reference drugs except **27**, **30**, and **31**.

The most sensitive fungi appeared to be *T. viride*, while *P. cyclpoium var verucosum* was the most resistant. 

As in case of bacteria fungi too showed different sensitivity towards compounds tested. Thus the order of activity of tested compounds against *T. viride* is: **3** = **4** = **28** > **1** > **2** = **5** = **11** = **15** = **6** = **19** > **7** = **9** = **10** = **18** > **29** > **13** > **20** = **26** > **12** = **14** = **22** = **24** = **25** > **8** > **17** > **21** = **31** > **27** = **30** > **6** > **23**, while for *P. v.c* the order is: **28** > **10** = **19** > **15** = **29** > **1** = **3** > **16** > **10** > **2** = **7** = **12** = **13** = **14** > **22** > **4** = **5** = **8** = **9** = **18** > **17** = **21** = **24** = **26** > **20** = **23** = **25** > **27** > **6** > **31** > **30.**

Different behavior was observed for *Aspergilus* species. Thus, the order of activity against *A. fumigatus* can be presented as follows: **16** = **18** = **19** > **1** = **3** = **4** = **9** = **10** = **11** = **15** = **20** = **2** = **22** = **23** = **24** = **28** > **5** = **12** = **13** = **14** = **17** = **25** = **29** > **2** > **7** = **8** = **27** > **6** > **31** > **30**, while against *A. versicolor* was: **19** > **28** > **1** = **15** = **17** = **23** = **16** > **3** = **4** = **5** = **8** = **10** = **11** = **22** > **9** = **24** > **18** = **20** > **2** = **6** = **12** = **13** = **14** = **23** = **29** > **7** > **21** > **26** > **27** > **31** > **30**.

Again, it was observed that many compounds exhibited the same sensitivity against the same fungi. For example, compounds **3**, **4, 9**–**11, 15, 20**–**24**, and **28** exhibited the same moderate activity against *A. fumigatus*.

Compounds **16**, **18** and **19** showed very good activity against *A. fumigatus* with MIC at 0.007–0.075 mg/mL and MFC at 0.015–0.15 mg/mL. A good activity was observed for **19** against *A. versicolor*. Compound **16** exhibited promising activity against *A. ochraceus*, while compound **3** and **28** against *P. funiculosum and P. ochrochloron* with MIC 0.007mg/mL and MFC 0.015 mg/mL. Very potent appeared to be compounds **3** and **28** as well as **4** against *T. viride* with MIC and MFC at 0.003 mg/mL and 0.007 mg/mL. The good activity was shown by compound **1** against *A. fumigatus* and *A. versicolor* and compounds **10** and **11** against *P. funiculosum* with MIC and MFC at 0.015 mg/mL and 0.037 mg/mL, respectively. 

The analysis of the structure-activity relationship revealed that the presence of 17α-aza- and 3β-hydroxy groups in d-homo-androst-5-en-17-one core (**28**) was the most beneficial for antifungal activity followed by the 17β-amino- on 5α-androst-2-en moiety **(19)**. On the contrary with antibacterial activity substitution of the 17-amino group appeared to be responsible for good activity. Thus, the presence of 17β-(*Ν*,*N*-dimethylamino)-as well as 17β-aminoethylamino substitution resulted in compounds **3** and **11**, which are among five the most active. The 17β-amino- (**1**), as well as 3-hydroximino substitution (**15**) of 5α-androstan-17β-ol core, also had a positive impact on antifungal activity.

The most negative effect on antifungal activity had the 17β-hydroxy group in 3α-aza-A-homoandrost-4-en-3-one (**30**).

### 2.4. Docking to Antibacterial Targets 

To elucidate the probable mechanism of antibacterial activity of tested compounds, docking studies were performed on five bacterial targets including DNA Topo IV, DNA Gyrase, *E. coli* Primase, Thymidylate kinase, and *E. coli* MurB enzymes. The obtained results are given in Table 5. 

Docking studies revealed that the scoring function associated with the free energy of binding to *E. coli* UDP-*N*-acetylenolpyruvoylglucosamine reductase (MurB) was lower than those obtained for the other enzymes. Hence, it may be concluded that *E. coli* MurB is the putative target responsible for the antibacterial activity of the tested compounds.

**Figure 1:**
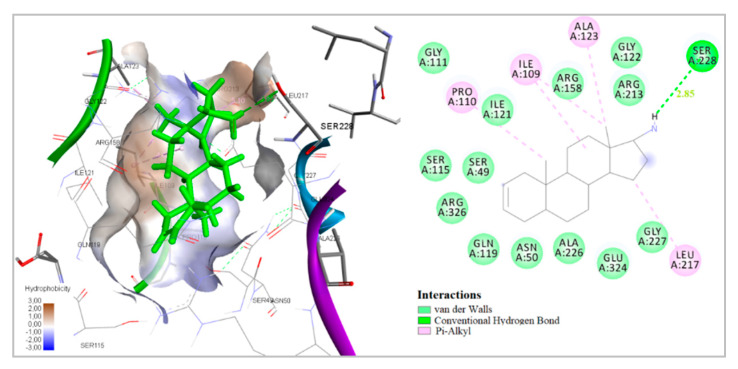
Docked conformation of the most active compound **19** in *E. coli* MurB.

The binding mode of the most active compound **19** (Est. binding energy: 9.62kcal/mol) (Figure 1) showed one hydrogen bond formed between the hydrogen atom of the NH_2_ group and the oxygen atom of the side chain of Ser228 (distance 2.53 A). The fused rings interact hydrophobically with the residues Arg213, Gly122, Arg158, Ala123, Ile109, Ile121, Pro110, Ser49, Arg326, Gln119, Asn50, Ala226, Glu324, and Leu217. 

### 2.5. Docking to Antifungal Targets

All the synthesized compounds and reference drugs were docked to different antifungal targets (Squalene synthase, Dihydrofolate reductase, and of *C. albicans*). It was found that the enzyme lanosterol 14α-demethylase of *C. albicans* was the most suitable for antifungal activity (Table 6) since the free binding energy was the lowest.

**Figure 2:**
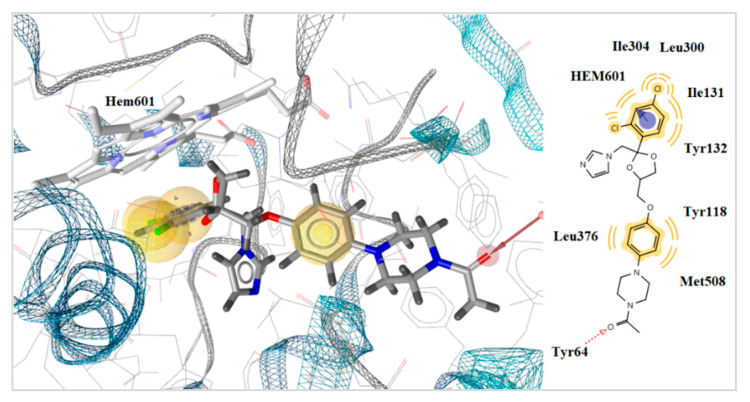
Docked conformation of ketoconazole in lanosterol 14α-demethylase of *C*. *albicans* (CYP51_ca_).

**Figure 3:**
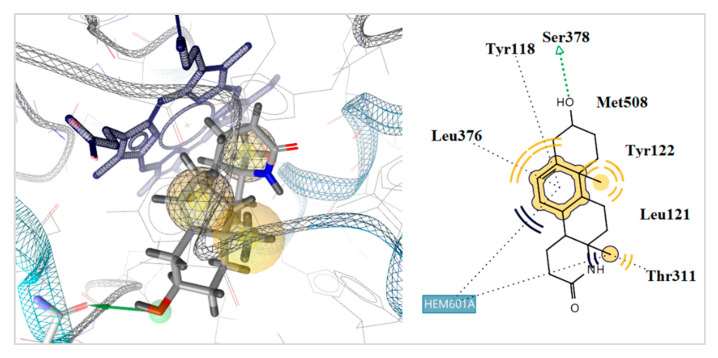
Docked conformation of the compound **28** in lanosterol 14α-demethylase of *C*. *albicans*.

**Figure 4:**
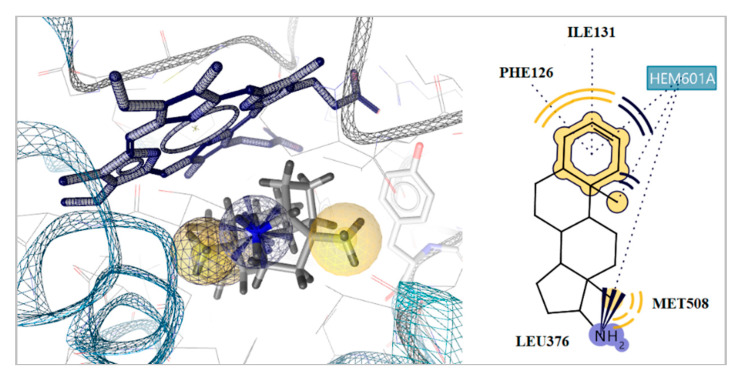
Docked conformation of the compound **19** in lanosterol 14α-demethylase of *C*. *albicans*.

Docking results showed that all the synthesized compounds may bind to CYP51Ca in a way that is similar to the binding of ketoconazole (Figure 2). The best docking score was calculated for compound **28**, which appeared to be the most favorable inhibitor experimentally. The docking pose of this compound is represented in Figure 3. Based on the docking results, compound **28** takes place inside the enzyme alongside to heme group, forming a hydrogen bond interaction between the oxygen atom of -OH substituent and the hydrogen atom of the side chain of the residue Ser378 (distance 1.98 Å). Moreover, fused rings interact hydroponically with the residues Tyr118, Leu121, Thr122, Leu376, Thr311, Met508, as well as with the heme group (Figure 4). In the case of compound **19**, docking scores revealed that it forms plenty of hydrophobic interactions. Furthermore, **19** forms positive ionizable interactions between the heme group and the -NH2 substituent (Figure 4), which stabilized more the complex of the ligand with the enzyme. This interaction is probably responsible for the lower free energy of binding compare to other compounds and ketoconazole.

## 3. Materials and Methods

### 3.1. Antimicrobial and Cytotoxic Activity Prediction

Prediction of the general antimicrobial activity was carried out using PASS (Prediction of Activity Spectra for Substances) software [47,48]. PASS uses structure–activity relationships derived from the data on biological activity of more than one million molecules, including twenty thousand with antibacterial and five thousand with antifungal activity; to classify previously unseen structures of chemical compounds as belonging or not belonging to one or more of the 5066 biological activity classes. PASS takes the chemical structure(s) of the molecule(s) under study as MDL MOL file or SDF (structure-data file) as input value and outputs the list of activities with corresponding assessments: Pa, assessment of probability for the structure to represent active molecule, and Pi, assessment of probability for the structure to represent inactive molecule.

The probable action of the studied compounds on the distinct microbial species and strains was estimated using web applications AntiBac-Pred [49,50] and AntiFun-Pred [51]. These tools are based on PASS and provide, in addition to its capabilities, the novel bioactivity data and web interface, also they are free to use. AntiBac-Pred allows to evaluate chemical compounds against 353 bacterial strains, and AntiFun-Pred, against 38 fungi. The results of the prediction are provided in a similar manner to that used in PASS. However, instead of the Pa and Pi values, only their difference is provided. The higher the value, the higher the confidence that compound will show activity.

CLC-Pred [56,57] is another PASS-based web application, which allows to predict cytotoxicity for chemical compounds against tumor and non-tumor cell lines. This tool was used to assess the potential cytotoxic effect of the chemical compounds under study.

### 3.2. Biological Evaluation

#### 3.2.1. Antibacterial Activity

The following Gram-negative bacteria were used: *Escherichia coli* (ATCC 35210), *Enterobacter cloacae, Pseudomonas aeruginosa* (ATCC 27853), *Salmonella typhimurium* (ATCC 13311), and the following Gram-positive bacteria: *Listeria monocytogenes* (NCTC 7973), *Bacillus cereus* (clinical isolate), *Micrococcus flavus* (ATCC 10240), and *Staphylococcus aureus* (ATCC 6538). The organisms were obtained from the Mycological Laboratory, Department of Plant Physiology, Institute for Biological Research “Siniša Stankovic”, Belgrade, Serbia.

The antibacterial assay was carried out by the microdilution method [57] in order to determine the antibacterial activity of compounds tested against the the above strains of human pathogenic bacteria. Compounds were diluted in DMSO, which was used as negative control (5%).

The bacterial suspensions were adjusted with sterile saline to a concentration of 1.0 × 10^−5^ cfu/mL. The innocula were prepared daily and stored at +4 °C until use. Dilutions of the innocula were cultured on solid medium to verify the absence of contamination and to check the validity of the inoculum [58,59].

##### Microdilution Test

The minimum inhibitory and bactericidal concentrations (MICs and MBCs) were determined using 96-well microtiter plates. The bacterial suspension was adjusted with sterile saline to a concentration of 1.0 × 10^−5^ cfu/mL. Compounds to be investigated were dissolved in broth LB medium (100 μL) with bacterial inocula (1.0 × 10^−4^ cfu per well) to achieve the wanted concentrations (1 mg/mL). The microplates were incubated for 24 h at 48 °C. The lowest concentrations without visible growth (under the binocular microscope) were defined as concentrations that completely inhibited bacterial growth (MICs). The compounds investigated were dissolved in 5% DMSO (1 mg/mL) and added in the LB medium to the inoculum. The MBCs were determined by serial sub-cultivation of 2 μL into microtiter plates containing 100 μL of broth per well and then submitted to further incubation for 72 h. The lowest concentration with no visible growth was defined as the MBC, indicating 99.5% killing of the original inoculum. The optical density of each well was measured at 655 nm by a Bio-Rad Laboratories Microplate Manager 4.0 and compared with a blank and the positive control. Streptomycin and ampicillin were used as positive controls (1 mg/mL) [58,59]. All experiments were performed in duplicate and repeated three times.

#### 3.2.2. Antifungal Activity

For the antifungal bioassays, eight fungi were used: *Aspergillus niger* (ATCC 6275), *Aspergillus ochraceus* (ATCC 12066), *Aspergillus fumigatus* (1022), *Aspergillus versicolor* (ATCC 11730), *Penicillium funiculosum* (ATCC 36839), *Penicillium ochrochloron* (ATCC 9112), *Trichoderma viride* (IAM 5061), and *Candida albicans* (human isolate). The organisms were obtained from the Mycological Laboratory, Department of Plant Physiology, Institute for Biological Research ‘‘Siniša Stankovic’’, Belgrade, Serbia.

The micromycetes were maintained on malt agar and the cultures stored at 4 °C and sub-cultured once a month. In order to investigate the antifungal activity of the extracts, a modified microdilution technique was used [51,52,53]. The fungal spores were washed from the surface of agar plates with sterile 0.85% saline containing 0.1% Tween 80 (*v*/*v*). The spore suspension was adjusted with sterile saline to a concentration of approximately 1.0 × 10^−5^ in a final volume of 100 μL per well. The innocula were stored at 4 °C for further use. Dilutions of the innocula were cultured on solid malt agar to verify the absence of contamination and to check the validity of the inoculum.

Minimum inhibitory concentration (MIC) determinations were performed by a serial dilution technique using 96-well microtiter plates. The compounds investigated were dissolved in 5% DMSO (1 mg/mL) and added in broth malt medium to the inoculum. The microplates were incubated for 72 h at 28 °C, respectively. The lowest concentrations without visible growth (under the binocular microscope) were defined as MICs.

The fungicidal concentrations (MFCs) were determined by serial subcultivation of a 2 mL into microtiter plates containing 100 μL of broth per well and then submitted to further incubation for 72 h at 28 °C.

The lowest concentration with no visible growth was defined as MFC, indicating 99.5% killing of the original inoculum. DMSO was used as a negative control; commercial fungicides, bifonazole and ketoconazole were used as positive controls (1–3000 mg/mL). All experiments were performed in duplicate and repeated three times.

### 3.3. Docking Studies

Τhe AutoDock 4.2^®^ (version 4.2.6, San Diego, California, CA, U.S.A) software was used for the docking simulation. The free energy of binding (ΔG) of DNA topoisomerase IV, *E. coli* primase, *E. coli* DNA GyrB, *E. coli* MurB, Thymidylate kinase, Squalene synthase, Dihydrofolate reductase and CYP51 of *C*. *albicans* in complex with the inhibitors were generated using this molecular docking program. The X-ray crystal structures data of all the enzymes used were obtained from the Protein Data Bank (PDB ID: 1S16, 1DDE, AKZN, AQGG, 2Q85, 1EZF, 4HOF, and 5V5Z, respectively). All procedures were performed according to our previous papers [60].

## 4. Conclusions

Thirty-one compounds were studied for antimicrobial activity in silico using PASS software as well as freely available web-services AntiBAC Pred, MICF Pred, and CLC-Pred. PASS predicted antibacterial activity for 27 of 31 molecules, and antifungal activity was predicted for 25 of 31 compounds with relatively low probability. Such a result leads us to the suggestion that the analyzed compounds are structurally different from well-known antimicrobial agents. Therefore, the studied compounds may be active against the resistant strains. Prediction of antibacterial and antifungal action on particular microbial strains with AntiBAC Pred and MICF Pred web-services demonstrated that the compounds may exhibit rather broad spectra of antimicrobial activities. GUSAR predicted rather low general toxicity for all compounds. CLC-Pred provided estimates that allow selecting the compounds with low probability of cytotoxicity for further studies. Therefore, testing of the antimicrobial activity against different microbial species for compounds with low chance of general toxicity and cytotoxicity looks reasonable.

The evaluation of the antibacterial activity of the tested compounds revealed that these molecules exhibit a significant pharmacological potential, having higher in vitro potency than the approved antibacterial drugs: Ampicillin and Streptomycin. In particular, studied compounds were more active against the resistant bacterial *E. coli*, and *P. aeruginosa* strains as well as methicillin-resistant *Staphylococcus aureus*. It should be mentioned that in general Gram-positive bacteria are more sensitive to the tested compounds than Gram-negative bacteria.

The presence of 17α–amino-3α-hydroxy, as well as 17β-amino-3β-hydroxy groups in 5α-androstan core was found to be beneficial for antibacterial activity whereas the presence of 17β-tosyloxy- as well as 3α- and 3β-azido groups was detrimental on activity.

Compounds’ antifungal effect (MIC at 0.007–0.45 mg/mL and MFC at 0.075–0.60 mg/mL) appeared to be superior to Ketoconazole and Bifonazole, which are widely used in clinical practice. The most sensitive fungi appeared to be *T. viride*, while *P. cyclpoium var verucosum* was the most resistant. 

The presence of 17α-aza- and 3β-hydroxy groups in d-homo-androst-5-en-17-one core (28) was the most beneficial for antifungal activity followed by the 17β-amino- on 5α-androst-2-en moiety.

Despite that, all compounds exhibited good activity against all bacteria and fungi tested, their sensitivity towards compounds, in general, was different.

The molecular docking analysis indicated that the putative mechanism of antibacterial activity is probably the inhibition of the *E. coli* MurB enzyme. 

Docking analysis to 14α-lanosterol demethylase (CYP51) and tetrahydrofolate reductase of *Candida albicans* indicated a probable implication of CYP51 reductase in the anti-fungal activity of the compounds.

## Figures and Tables

**Table 1:** Structure of compounds.

N	Structure	N	Structure
**1**	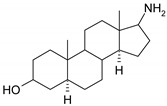	**17**	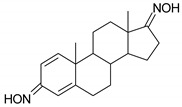
**2**	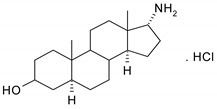	**18**	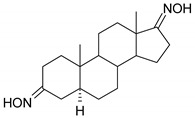
**3**	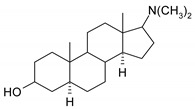	**19**	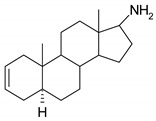
**4**	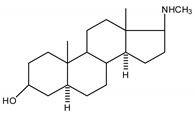	**20**	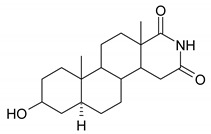
**5**	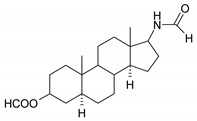	**21**	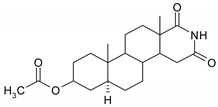
**6**	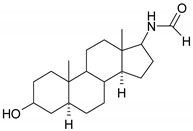	**2** **2**	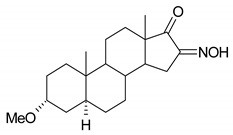
**7**	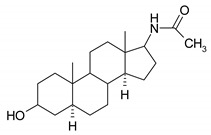	**2** **3**	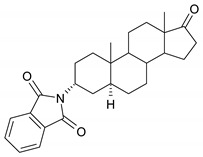
**8**	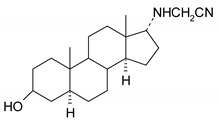	**2** **4**	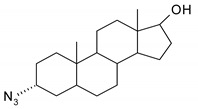
**9**	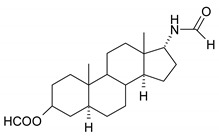	**25**	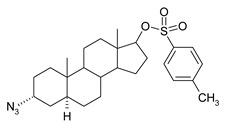
**10**	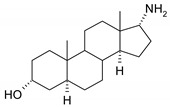	**26**	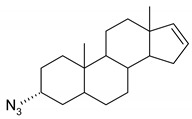
**11**	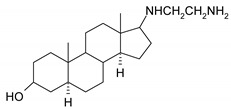	**27**	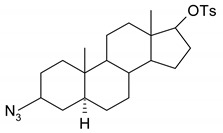
**12**	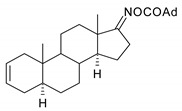	**28**	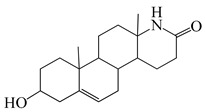
**13**	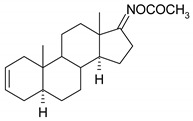	**29**	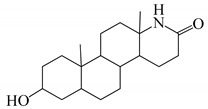
**14**	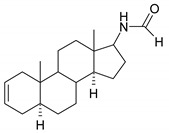	**30**	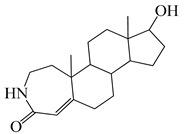
**15**	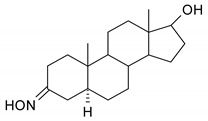	**31**	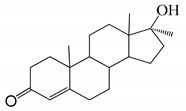
**16**	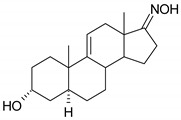		

**Table 2:** Predicted biological activity spectra for the studied molecules.

N	Antibacterial Pa	Antifungal Pa	N	Antibacterial Pa	Antifungal Pa
**1**	0.420	0.350	**16**	0.276	0.375
**2**	0.420	0.350	**17**	0.351	-
**3**	0.370	0.349	**18**	0.227	0.256
**4**	0.340	0.342	**19**	0.208	0.253
**5**	0.374	0.180	**20**	0.320	0.248
**6**	0.437	0.308	**21**	0.288	0.243
**7**	0.334	0.231	**22**	0.299	0.336
**8**	0.315	0.188	**23**	0.348	-
**9**	0.374	0.180	**24**	0.285	0.286
**10**	0.420	0.350	**25**	0.278	0.171
**11**	0.438	0.368	**26**	0.340	0.185
**12**	0.239	0.203	**27**	0.278	0.171
**13**	0.199	0.214	**28**	-	-
**14**	0.458	0.216	**29**	-	-
**15**	0.376	0.427	**30**	-	-
			**31**	-	-

**Table 3:** Antibacterial activity of compounds 1–31 (MIC and MBC in mg/mL).

Compounds		*S. a.*	*MRSA*	*L. m.*	*P. a.*	PaO1	*E. coli*	*E. coli res*	*S. thy.*
**1**	**MIC**	0.010	0.010	0.025	0.037	0.025	0.025	0.025	0.025
	**MBC**	0.015	0.015	0.037	0.075	0.037	0.037	0.037	0.037
**2**	**MIC**	0.025	0.025	0.050	0.025	0.015	0.075	0.050	0.050
	**MBC**	0.037	0.037	0.075	0.037	0.075	0.15	0.075	0.075
**3**	**MIC**	0.010	0.007	0.020	0.30	0.15	-	0.020	0.020
	**MBC**	0.015	0.015	0.037	0.45	0.30	-	0.037	0.037
**4**	**MIC**	0.020	0.020	0.050	0.015	0.15	0.20	0.020	0.020
	**MBC**	0.037	0.037	0.075	0.037	0.30	0.30	0.037	0.037
**5**	**MIC**	0.20	0.050	0.10	0.050	-	-	0.10	0.10
	**MBC**	0.30	0.037	0.15	0.075	-	-	0.15	0.15
**6**	**MIC**	-	-	-	-	-	-	0.30	-
	**MBC**	-	-	-	-	-	-	0.60	-
**7**	**MIC**	-	0.075	0.20	0.20	0.15	0.10	0.15	0.20
	**MBC**	-	0.15	0.30	0.30	0.30	0.15	0.30	0.30
**8**	**MIC**	0.30	0.037	0.20	0.15	0.15	0.075	0.15	0.20
	**MBC**	0.45	0.075	0.15	0.30	0.30	0.15	0.30	0.30
**9**	**MIC**	-	0.050	0.30	0.20	0.10	0.10	0.15	0.30
	**MBC**	-	0.075	0.60	0.30	0.15	0.15	0.30	0.45
**10**	**MIC**	0.020	0.020	0.020	0.037	0.10	0.010	0.015	0.020
	**MBC**	0.037	0.037	0.037	0.075	0.15	0.015	0.037	0.037
**11**	**MIC**	0.050	0.005	0.10	0.020	0.10	0.050	0.037	0.10
	**MBC**	0.075	0.007	0.15	0.037	0.15	0.075	0.075	0.15
**12**	**MIC**	-	0.10	0.30	0.20	0.10	0.20	0.15	-
	**MBC**	-	0.15	0.60	0.30	0.15	0.30	0.30	-
**13**	**MIC**	-	0.037	-	0.15	0.15	0.10	0.15	-
	**MBC**	-	0.075	-	0.30	0.30	0.20	0.30	-
**14**	**MIC**	0.20	0.020	0.20	0.10	0.15	0.10	0.15	0.20
	**MBC**	0.30	0.037	0.30	0.15	0.30	0.15	0.30	0.30
**15**	**MIC**	-	0.020	0.15	0.10	0.10	0.060	0.15	0.20
	**MBC**	-	0.040	0.30	0.15	0.15	0.080	0.30	0.30
**16**	**MIC**	0.20	0.030	0.15	0.08	0.10	0.04	0.08	0.15
	**MBC**	0.30	0.040	0.30	0.15	0.15	0.08	0.15	0.30
**17**	**MIC**	-	0.20	0.30	0.15	-	0.15	-	0.30
	**MBC**	-	0.30	0.60	0.30	-	0.30	-	0.60
**18**	**MIC**	0.30	0.075	0.30	0.10	0.10	0.20	-	0.30
	**MBC**	0.60	0.15	0.60	0.15	0.15	0.30	-	0.60
**19**	**MIC**	0.0005	0.00015	0.0015	0.0015	0.015	0.007	0.003	0.005
	**MBC**	0.0007	0.0003	0.003	0.003	0.037	0.015	0.007	0.007
**20**	**MIC**	0.20	0.075	0.075	0.020	0.15	0.20	0.15	0.15
	**MBC**	0.30	0.15	0.30	0.037	0.30	0.30	0.30	0.30
**21**	**MIC**	-	0.075	0.30	-	-	-	0.10	0.30
	**MBC**	-	0.15	0.45	-	-	-	0.15	0.60
**22**	**MIC**	0.10	0.0037	0.10	0.075	0.050	0.037	0.015	0.15
	**MBC**	0.15	0.015	0.15	0.15	0.075	0.075	0.037	0.30
**23**	**MIC**	0.20	0.037	0.20	0.10	0.20	0.05	0.15	0.20
	**MBC**	0.30	0.075	0.30	0.20	0.30	0.075	0.30	0.30
**24**	**MIC**	0.30	0.05	0.20	0.075	0.050	0.05	0.007	0.20
	**MBC**	0.45	0.075	0.30	0.15	0.075	0.075	0.037	0.30
**25**	**MIC**	-	0.15	-	-	-	-	0.10	-
	**MBC**	-	0.30	-	-	-	-	0.15	-
**26**	**MIC**	-	0.037	-	0.075	0.15	0.075	0.075	-
	**MBC**	-	0.075	-	0.15	0.30	0.15	0.15	-
**27**	**MIC**	-	0.30	-	-	-	0.30	-	-
	**MBC**	-	0.60	-	-	-	0.60	-	-
**28**	**MIC**	0.15	0.037	0.075	0.02	0.007	0.02	0.037	0.15
	**MBC**	0.30	0.075	0.15	0.037	0.015	0.075	0.075	0.30
**29**	**MIC**	0.45	0.05	0.075	0.02	0.05	0.05	0.30	0.15
	**MBC**	0.60	0.075	0.15	0.037	0.075	0.075	0.45	0.30
**30**	**MIC**	0.20	0.10	0.075	0.075	0.037	0.037	0.075	0.10
	**MBC**	0.30	0.15	0.15	0.15	0.075	0.075	0.15	0.15
**31**	**MIC**	0.45	0.20	0.20	0.10	0.05	0.10	0.15	0.45
	**MBC**	0.60	0.30	0.30	0.15	0.075	0.15	0.30	0.60
**Streptomycin**	**MIC**	0.10	0.10	0.15	0.10	0.05	0.10	0.10	0.10
	**MBC**	0.20	-	0.30	0.20	0.1	0.20	0.20	0.20
**Ampicillin**	**MIC**	0.10	-	0.15	0.30	0.2	0.15	0.20	0.10
	**MBC**	0.15	-	0.30	0.50	-	0.20	-	0.20

“-”—no activity; *S. a.*—*S. aureus*; MRSA—methicillin resistant *S. aureus*; *L. m.*—*L. monocytogenes*; *P. a.*—*P. aeruginosa*; *P.aO1*—*P. aeruginosa* resistant; *E. c.*—*E.coli*; *S. t.*—*S. typhimurium*; Amp.—Ampicillin; Strept.—Streptomycin; Relative standard deviations were all < 2.0.

**Table 4:** Antifungal activity of compounds 10–31 (MIC and MFC in mg/mL).

Compounds		*A. fum.*	*A. v.*	*A. o.*	*A. n.*	*T. v.*	*P. f.*	*P. o.*	*P. v.c.*
**1**	**MIC**	0.037	0.015	0.015	0.037	0.007	0.037	0.007	0.037
	**MFC**	0.075	0.037	0.037	0.075	0.010	0.075	0.015	0.075
**2**	**MIC**	0.10	0.075	0.075	0.15	0.007	0.075	0.15	0.075
	**MFC**	0.15	0.15	0.15	0.30	0.015	0.15	0.30	0.15
**3**	**MIC**	0.037	0.037	0.037	0.007	0.003	0.007	0.007	0.015
	**MFC**	0.075	0.075	0.075	0.015	0.007	0.015	0.015	0.037
**4**	**MIC**	0.037	0.037	0.037	0.075	0.003	0.075	0.037	0.15
	**MFC**	0.075	0.075	0.075	0.15	0.007	0.15	0.075	0.30
**5**	**MIC**	0.075	0.037	0.037	0.15	0.007	0.037	0.075	0.15
	**MFC**	0.15	0.075	0.075	0.30	0.015	0.075	0.15	0.30
**6**	**MIC**	0.45	0.075	0.30	0.15	0.45	0.20	0.45	0.45
	**MFC**	0.60	0.15	0.45	0.30	0.60	0.45	0.60	0.60
**7**	**MIC**	0.30	0.15	0.10	0.020	0.015	0.10	0.075	0.075
	**MFC**	0.60	0.30	0.15	0.037	0.037	0.15	0.15	0.15
**8**	**MIC**	0.30	0.037	0.020	0.075	0.10	0.05	0.075	0.15
	**MFC**	0.60	0.075	0.037	0.15	0.15	0.075	0.15	0.30
**9**	**MIC**	0.037	0.05	0.020	0.037	0.015	0.037	0.075	0.15
	**MFC**	0.075	0.075	0.037	0.075	0.037	0.075	0.15	0.30
**10**	**MIC**	0.037	0.015	0.015	0.075	0.015	0.015	0.015	0.015
	**MFC**	0.075	0.037	0.037	0.15	0.037	0.037	0.037	0.037
**11**	**MIC**	0.037	0.037	0.037	0.007	0.007	0.015	0.015	0.075
	**MFC**	0.075	0.075	0.075	0.015	0.015	0.037	0.037	0.015
**12**	**MIC**	0.075	0.075	0.037	0.015	0.05	0.075	0.15	0.075
	**MFC**	0.15	0.15	0.075	0.037	0.075	0.15	0.30	0.15
**13**	**MIC**	0.075	0.075	0.05	0.005	0.037	0.075	0.15	0.075
	**MFC**	0.15	0.15	0.075	0.015	0.05	0.15	0.30	0.15
**14**	**MIC**	0.075	0.075	0.037	0.037	0.05	0.075	0.075	0.075
	**MFC**	0.15	0.15	0.075	0.075	0.075	0.15	0.15	0.15
**15**	**MIC**	0.037	0.015	0.015	0.037	0.007	0.015	0.02	0.05
	**MFC**	0.075	0.037	0.037	0.075	0.015	0.075	0.037	0.037
**16**	**MIC**	0.015	0.015	0.007	0.037	0.007	0.015	0.037	0.05
	**MFC**	0.037	0.075	0.015	0.075	0.015	0.037	0.075	0.075
**17**	**MIC**	0.075	0.015	0.075	0.15	0.10	0.15	0.20	0.20
	**MFC**	0.15	0.037	0.15	0.30	0.15	0.30	0.30	0.30
**18**	**MIC**	0.015	0.037	0.10	0.075	0.015	0.15	0.075	0.15
	**MFC**	0.037	0.15	0.15	0.15	0.037	0.30	0.15	0.30
**19**	**MIC**	0.015	0.007	0.015	0.015	0.007	0.037	0.037	0.015
	**MFC**	0.037	0.015	0.037	0.070	0.015	0.070	0.070	0.037
**20**	**MIC**	0.075	0.037	0.15	0.30	0.075	0.15	0.30	0.30
	**MFC**	0.15	0.15	0.30	0.45	0.15	0.30	0.45	0.45
**21**	**MIC**	0.075	0.075	0.30	0.30	0.15	0.10	0.20	0.20
	**MFC**	0.15	0.30	0.45	0.60	0.30	0.15	0.30	0.30
**22**	**MIC**	0.037	0.037	0.075	0.075	0.050	0.075	0.15	0.10
	**MFC**	0.075	0.075	0.15	0.15	0.075	0.15	0.30	0.15
**23**	**MIC**	0.037	0.015	-	0.037	-	0.015	0.037	0.30
	**MFC**	0.075	0.037	-	0.075	-	0.037	0.075	0.45
**24**	**MIC**	0.037	0.050	0.050	0.075	0.050	0.075	0.10	0.20
	**MFC**	0.075	0.075	0.075	0.15	0.075	0.15	0.15	0.30
**25**	**MIC**	0.075	0.075	0.037	0.10	0.050	0.050	0.30	0.30
	**MFC**	0.15	0.15	0.075	0.15	0.075	0.075	0.45	0.45
**26**	**MIC**	0.075	0.15	0.075	0.10	0.037	0.075	0.075	0.20
	**MFC**	0.15	0.30	0.15	0.15	0.75	0.15	0.15	0.30
**27**	**MIC**	0.30	0.30	0.30	0.60	0.20	0.30	0.30	0.30
	**MFC**	0.60	0.45	0.45	0.90	0.30	0.45	0.60	0.60
**28**	**MIC**	0.037	0.007	0.015	0.037	0.003	0.007	0.007	0.007
	**MFC**	0.075	0.015	0.037	0.075	0.007	0.015	0.015	0.015
**29**	**MIC**	0.075	0.075	0.037	0.007	0.020	0.05	0.020	0.050
	**MFC**	0.15	0.15	0.075	0.015	0.037	0.075	0.037	0.037
**30**	**MIC**	2.40>	0.60	1.20	2.40	0.20	0.60	0.30	1.20
	**MFC**	2.40>	1.20	1.80	2.40	0.30	1.20	0.60	2.40
**31**	**MIC**	1.00	0.20	0.20	0.60	0.15	0.60	0.45	0.60
	**MFC**	1.80	0.60	0.30	1.20	0.30	1.20	1.20	1.80
**Ketoconazole**	**MIC**	0.20	0.20	0.15	0.20	1.00	0.20	1.00	0.20
	**MFC**	0.50	0.50	0.20	0.50	1.50	0.50	1.50	0.30
**Bifonazole**	**MIC**	0.15	0.10	0.15	0.15	0.15	0.20	0.10	0.10
	**MFC**	0.20	0.20	0.20	0.20	0.20	0.25	0.25	0.20

*A. fum.—A. fumigatus; A. v.—A. versicolor; A. o.—A. ochraceus; A. n.—A. niger; T. v.—T. viride; P. f.—P. funiculosum; P. o.—P. ochrochloron; C. a.—C. albicans; P. v.c.—P. cyclpoium var verucosum.* Relative standard deviations were all < 2.20, except for antimycotics < 4.50.

**Table 5:** Molecular docking binding affinities.

N/N	Est. Binding Energy (kcal/mol)					Binding Affinity Score *E. coli* MurB	I-H	Residues *E. coli* MurB
	DNA Topo IVPDB ID: 1S16	*E. coli* Primase PDB ID: 1DDE	Gyrase PDB ID: 1KZN	Thymidylate Kinase PDB ID: 4QGG	*E. coli* MurB PDB ID: 2Q85			
1	−5.02	-	−7.22	−2.88	−9.12	−31.47	1	Ser228
2	−4.96	−1.86	−7.03	-	−8.73	−30.54	2	Tyr189, Asn232
3	-	-	−6.14	−2.01	−8.45	−29.48	2	Asn232, Glu324
4	−3.82	−1.63	−6.85	-	−8.71	−30.12	1	Ser228
5	−2.18	-	−5.73	−2.15	−7.70	−27.56	2	Tyr124, Arg213
6	-	-	-	-	−5.14	−17.24	-	-
7	-	-	−7.43	-	−7.02	−25.31	1	Gly122
8	−1.21	-	−3.31	-	−6.50	−22.13	1	Arg158
9	-	-	−2.88	-	−6.25	−21.01	1	Arg213
10	−5.22	−2.35	−7.31	−2.89	−9.36	−31.58	1	Ser228
11	−3.01	-	−6.16	−2.13	−8.70	−29.55	1	Ser228
12	-	-	-	−1.93	−6.57	−22.43	1	Arg158
13	−1.25	-	−3.38	-	−6.62	−22.79	1	Asn232
14	-	−1.13	−5.05	-	−7.22	−25.41	1	Tyr189
15	−2.31	-	−5.02	-	−7.71	−27.58	2	Arg213, Asn232
16	−2.11	-	−4.81	-	−7.69	−27.55	2	Arg213, Asn232
17	-	-	−2.87	-	−6.22	−21.00	1	Arg213
18	−3.55	-	−2.31	-	−6.03	−19.25	1	Asn232
19	−5.24	−2.88	−7.36	−2.63	−9.62	−31.89	1	Ser228
20	-	−1.32	−4.16	−1.29	−7.51	−26.31	1	Asn232
21	-	-	-	-	−5.21	−18.92	-	-
22	−1.25	−2.96	−6.08	-	−8.11	−28.74	1	Ser228
23	-	-	−4.88	-	7.53	−26.42	1	Arg213
24	-	-	−3.52	-	−6.85	−24.16	1	Arg213
25	-	-	-	-	−4.71	−15.03	-	-
26	−2.63	-	−6.22	-	−8.13	−28.74	1	Ser228
27	-	-	-	-	−3.29	−11.24	-	-
28	-	−1.23	−6.23	-	−8.27	−28.96	1	Ser228
29	-	−1.96	−5.27	-	−7.23	−25.44	1	Gly122
30	-	-	−6.05	-	−8.24	−28.53	1	Ser228
31	-	-	−1.09	-	−1.02	−3.64	-	-

**Table 6:** Molecular docking binding affinities.

Com.		Est. Binding Energy (kcal/mol)			Binding Affinity Score CYP51 of *C. albicans* PDB ID: 5V5Z	I-H	Residues CYP51 of *C. albicans* PDB ID: 5V5Z	Interaction with Heme
		Squalene SynthasePDB ID:1EZF	Dihydrofolate ReductasePDB ID: 4HOF	CYP51 of *C. albicans* PDB ID: 5V5Z				
	1	−1.85	−6.92	−9.31	−30.74	1	Ser312	Hem601
	2		−4.21	−7.60	−26.19	2	Tyr118, Ser312	
	3	−3.02	−7.66	−9.79	−32.86	1	Tyr118	Hem601
	4		−6.11	−8.64	−28.19	-	-	Hem601
	5		−5.73	−7.91	−26.28	-	-	Hem601
	6			−6.32	−22.41	1	Tyr132	
	7		−3.85	−7.13	−26.04	-	-	Hem601
	8		−3.33	−7.01	−25.97	-	-	Hem601
	9	−1.06	−6.30	−8.73	−28.41	-	-	Hem601
	10	−1.25	−6.54	−8.80	−29.13	-	-	Hem601
	11	−3.25	−7.45	−9.72	−32.67	1	Tyr118	Hem601
	12		−5.88	−8.32	−27.53	-	-	Hem601
	13			−4.12	−9.01	-	-	
	14		−5.93	−8.37	−27.16	-	-	Hem601
	15	−2.84	−7.23	−9.51	−31.93	-	-	Hem601
	16	−1.86	−6.95	−9.33	−30.82	1	Ser312	Hem601
	17	−1.12	−2.47	−7.02	−25.93	-	-	Hem601
	18		−4.22	−7.66	−26.21	2	Tyr118, Ser312	
	19	−3.11	−8.01	−10.06	34.16	-	-	Hem601
	20			−6.71	−24.13	1	Tyr118	
	21			−6.55	−23.58	1	Tyr118	
	22		−5.44	−7.68	−26.13	2	Tyr118, Tyr132	
	23			−3.21	−7.12	-	-	
	24		−5.87	−8.34	−27.36	-	-	Hem601
	25			−7.02	−25.94	-	-	Hem601
	26	−1.03		−6.75	−24.19	-	-	Hem601
	27			−5.84	−19.05	-	-	
	28	−3.25	−8.17	−11.25	−34.58	1	Tyr378	Hem601
	29	−3.10	6.33	−8.81	−29.16	-	-	Hem601
	30			−4.82	−9.15	-	-	
	31	−1.59		−5.37	−11.28	-	-	
ketoconazole		-	−6.75	−8.23	−22.47	1	Tyr64	Hem601

## Supplementary Materials

The following are available online at https://www.mdpi.com/2079-6382/9/5/224/s1, Excel file with the prediction results for the studied thirty-one compounds with AntiBAC Pred and MICF Pred web-services (includes up to top three predicted bacteria and fungi with the estimated confidence values and hypertext links to the description of the species in the ChEMBL database) as well as the results obtained with and CLC-Pred web-service.
